# Extra-Pulmonary Complications in SARS-CoV-2 Infection: A Comprehensive Multi Organ-System Review

**DOI:** 10.3390/microorganisms10010153

**Published:** 2022-01-12

**Authors:** Taylor J. Louis, Ahmad Qasem, Latifa S. Abdelli, Saleh A. Naser

**Affiliations:** Division of Molecular Microbiology, Burnett School of Biomedical Sciences, College of Medicine, University of Central Florida, Orlando, FL 32816, USA; taylor.louis@knights.ucf.edu (T.J.L.); ahmadqasem@knights.ucf.edu (A.Q.); latifa.abdelli@ucf.edu (L.S.A.)

**Keywords:** SARS-CoV-2, COVID-19, infection, viruses, RAAS, ACE2

## Abstract

Coronavirus disease (COVID-19), caused by severe acute respiratory syndrome coronavirus 2 (SARS-CoV-2) infection, is typically presented with acute symptoms affecting upper and lower respiratory systems. As the current pandemic progresses, COVID-19 patients are experiencing a series of nonspecific or atypical extra-pulmonary complications such as systemic inflammation, hypercoagulability state, and dysregulation of the renin–angiotensin–aldosterone system (RAAS). These manifestations often delay testing, diagnosis, and the urge to seek effective treatment. Although the pathophysiology of these complications is not clearly understood, the incidence of COVID-19 increases with age and the presence of pre-existing conditions. This review article outlines the pathophysiology and clinical impact of SARS-CoV-2 infection on extra-pulmonary systems. Understanding the broad spectrum of atypical extra-pulmonary manifestations of COVID-19 should increase disease surveillance, restrict transmission, and most importantly prevent multiple organ-system complications.

## 1. Introduction

*Coronaviruses* are a large family of viruses known as *Orthocoronavirinae* that usually cause mild to moderate upper-respiratory tract illnesses, like the common cold [1]. Over the last few decades, the coronavirus family has been the origin of multiple highly infectious global outbreaks. The most significant ones were the 2003 Severe Acute Respiratory Syndrome coronavirus-1 (SARS-CoV-1) outbreak in China, and the 2012 Middle East Respiratory Syndrome coronavirus (MERS-CoV) outbreak in Saudi Arabia [1,2]. The most recent coronavirus outbreak developed in December 2019 is mainly characterized by signs of acute hypoxic respiratory failure and severe pneumonia [3]. Further genomic sequencing analysis revealed that the causative pathogenic agent of this outbreak is a positive-sense single-stranded RNA virus, which was later identified in February 2020 as the severe acute respiratory syndrome coronavirus 2 (SARS-CoV-2) by the International Virus Classification Commission [4]. The disease caused by SARS-CoV-2 is known as coronavirus disease 2019 (COVID-19) and has since become a global pandemic [5].

COVID-19-related deaths have exceeded its two predecessors, SARS-CoV-1 and MERS-CoV, combined [6]. The number of identified cases is steadily increasing, and the outbreak has rapidly spread to 222 countries over a short period of time [6]. As of 24 August 2021, 213 million cases and 4.4 million deaths have been documented worldwide [6]. Therefore, understanding the clinical complications of SARS-CoV-2 infection is pivotal. The clinical presentation of SARS-CoV-2 infection could range from asymptomatic or subclinical infection to severe pneumonia with respiratory failure and even death [7]. Around 80% of COVID-19 patients have mild to moderate disease, 15% develop severe disease, and about 5% end up in a critical condition that requires hospitalization [8]. Specifically, elderly patients with underlying chronic conditions such as cardiovascular complications, diabetes, and emphysema are more vulnerable to the development of severe disease condition, as well as death [9].

Most of COVID-19 patients are typically presented with fever, dry cough, dyspnea, sore throat, and fatigue [10]. Additionally, abdominal pain, vomiting, and diarrhea are considered less commonly reported symptoms [10]. However, some COVID-19 patients might experience nonspecific or atypical symptoms, leading to a delay in testing, diagnosis, and isolation [9]. The pathophysiology behind these atypical presentations is still poorly understood, but the possibility of experiencing these atypical symptoms increases with advanced age and pre-existing comorbidities [11].

The interaction between the SARS-CoV-2 spike protein (S) and angiotensin-converting enzyme 2 (ACE2) receptors facilitates viral entry into host cells [10]. Theoretically, any organ-system expressing ACE2 receptors is potentially susceptible to SARS-CoV-2 direct invasion, including the central nervous system, the gastrointestinal tract, the kidneys, the heart, and the reproductive system [12]. Besides, there are various indirect mechanisms of SARS-CoV-2 infection that may result in multiple organ-system consequences, such as systemic inflammation, hypercoagulability state, and dysregulation of the renin–angiotensin–aldosterone system (RAAS) [13].

The clinical manifestations of COVID-19 should be carefully monitored in clinical settings, in order to mitigate and prevent multiple organ-system complications by implementing organ-specific therapeutic approaches. In this review article, we discuss the pathophysiology and clinical impact of SARS-CoV-2 infection on various organ-systems, to provide a perspective on the major extra-pulmonary consequences of this viral infection.

## 2. Immunological Complications

SARS-CoV-2 is a pathogen with morphologically distinct crown-like projections [14,15]. It enters the host via fomite exposure, respiratory droplets, and infective aerosols [14,16]. Once inside the body, transmembrane serine protease 2 (TMPRSS2), furin, and cathepsin B/L cleave the spike proteins, thereby acquiescing ACE2 receptor binding [14,15,17]. These concerted processes facilitate viral fusion with the host cell membrane [15,17]. Ensuing, SARS-CoV-2 replicates until the cell lyses, resulting in systemic dissemination [14]. The average incubation period for SARS-CoV-2 is approximately 2–14 days [16]. During the prodromal phase, symptomatic adults may experience symptoms that typically last for 2 months, however, long hauler indicia have been reported [16].

Foregoing studies have demonstrated that the composition of immune cells is an effective determinant of disease severity [16]. Accordingly, clinicians may find it useful to quantify the ratio of neutrophils to lymphocytes in order to gauge the extent of systemic inflammation [16]. Inflammation is an essential prognosis factor for COVID-19 disease [16]. As such, comorbidities with extant low-grade inflammation such as obesity and advanced age predispose patients to a more severe infection [16]. Clinical trials have determined that timely administration of immunosuppressive drugs can significantly reduce mortalities amongst emergent patients [18]. However, the precarity of COVID-19 requires these treatments be used at the clinician’s discretion.

The generalization of COVID-induced morbidities is due in part to the ubiquity of ACE2 expression and production [16]. *ACE2* mRNA is abundant within the epithelial cells of the liver, intestines, kidneys, heart, and oral mucosa [16]. In addition, its protein distribution is abundant within the alveoli, although the tissue distribution within other organs may vary [16]. To that end, improving overall health with natural remedies may confer some advantage [16]. The World Health Organization (WHO) recommends individuals stay hydrated and consume foods high in vitamins B, C, D, and E [14]. Moreover, the WHO also recommends eating foods rich in zinc, iron, and protein to boost immunity [14]. These countermeasures may not cure coronavirus infection; however, they have the potential to ameliorate COVID-associated symptoms [14].

### 2.1. SARS-CoV-2-Induced Complement Activation

Once SARS-CoV-2 enters a cells, itinerant mannan-binding proteins (MBP) adhere to viral spike proteins, which sequester MBP-associated serine proteases 1 and 2 (MASPs 1/2) [19,20,21]. The association of MASPs 1/2 with affixed MBP initiates viral neutralization via the lectin complement pathway [19]. Alternatively, if a virion manages to escape opsonization and infects a host cell, MBP-like lectins will bind to oligosaccharides present on the virally infected cell surface to initiate cell lysis via the classical complement pathway [22]. The lysis of SARS-CoV-2 infected cells and itinerant virions release SARS-CoV-2 nucleocapsid (N) proteins into the extracellular space, which after dimerization, amplifies MASP 2 auto-activation, leading to hyperactivation of the lectin complement cascade [19]. Aberrant complement activation latterly engenders exorbitant release of anaphylatoxins C3a and C5a, which activates an assortment of immune cells and induces pro-inflammatory cytokine release [19]. Additionally, fragments C3b and C5b from the cytolytic terminal complex also induce the synthesis of pyrogenic metabolites such as thromboxane B2, prostaglandin E2, and leukotrienes, which aggravates inflammatory tissue damage and also facilitates leukocyte recruitment [19]. Recruited neutrophils will then amplify complement activation by secreting properdin, which evokes C5a secretion from adjacent neutrophils and enhances C3 tickover within the alternative complement pathway [23]. Collectively, these processes foment cyclic complement activation, which eventually incites hypercytokinemia, anti-neutrophil cytoplasmic autoantibody-associated vasculitis, immune paralysis, bystander injury to host cells, coagulopathies, acute respiratory distress syndrome (ARDS), and ultimately multiorgan failure and/or death [19,23,24]. Increased soluble C5a levels within patient sera and amassment of complement deposition within the skin and lungs are predictive markers for poorer clinical outcomes [25,26]. Therefore, clinicians may utilize complement suppressors to mitigate these sequelae [19,25].

According to anecdotal reports, emergent COVID-19 patients treated with Eculizumab (a C5 inhibitor) or AMY-101 (a C3 inhibitor) experienced better clinical outcomes than non-treated patients [23]. Other suggested therapies include stimulating or enhancing endogenous complement regulator proteins, blocking the interaction between N proteins and MASP 2, and/or disrupting N protein dimerization [19,23,24]. These therapies have been conceptualized in the literature but require further investigation to determine its clinical application [19,23,24]. Fortunately, complement activation and inflammasomes functionality are interconnected [24]. So, if complement activation was pacified, inflammasome activation would be better regulated [24,27].

### 2.2. The Innate Immune Response

The membrane attack complex (MAC) can incite NLRP3 inflammasome activation via IL-1β secretion and increased intracellular Ca^2+^ concentrations, following pore formation [28]. The activated inflammasome will in turn activate gasdermin-D, which engenders primary monocyte pyroptosis [29]. Normally, the complement factor C1q would modulate inflammasome activity by preventing caspase-1 cleavage [28]. However, complement activation continues unabated, resulting in severe Lymphopenia and reduced interferon-gamma (IFN-γ) secretion [16,29]. By suppressing the intrinsic IFN response, SARS-CoV-2 allots time to rev up replication, which results in the destruction of adjacent tissues [16]. Consequently, this engenders a fractious immune response, resulting in hypercytokinemia [16]. Curiously, age and sex significantly influence the robustness of the IFN response [16]. Males tend to develop more acute infections while females typically elicit exuberant IFN responses [16]. Females also develop stronger vaccine responses; garner bounteous IFN neutralizing autoantibodies; and display better survivability rates among emergent patients [16]. For this reason, researchers have speculated that the localization of the TLR7 gene on the X chromosome confers an advantage against coronavirus infection [16]. However, despite this sexual antagonism, females are more inclined to develop long haulers syndromes and adverse vaccine effects [16]. In like manner, these trends were also reflected within the young versus old populations.

The adolescent immune system is more adept at confronting unfamiliar challenges, whereas older immune systems heavily rely on immunological memory [16]. This is due, in part, to the age-related involution of the thymus [16]. In the interim of adulthood, the capacity of the thymus to release naïve T cells steadily declines; approximately 3% per year [16]. As a result, older individuals have a deficient IFN response, which may increase their susceptibility to contracting coronavirus infection [16]. Nevertheless, the latter statement seems to be more of a postulation than a verity because infants and toddlers also have a deficient IFN response and yet, concurrent studies have indicated their limited risk [16]. For this reason, researchers are investigating the efficacy of other predisposing factors.

Another age-related comorbidity is multisystem inflammatory syndrome (MIS-C) [16]. MIS-C is a hyperinflammatory malady, which manifests in children and young adults approximately 2–6 weeks post infection [16]. Although rare, MIS-C displays clinical features similar to Kawasaki disease [16]. However, the presence of heart and intestinal morbidities distinguishes MIS-C as the differential diagnosis [16]. Treatment regimens for MIS-C include steroids, anti-cytokine therapies, and intravenous immunoglobulin administration [16]. The pathogenesis of COVID-induced MIS-C is not fully understood [16].

### 2.3. The Adaptive Immune Response

**Figure 1:**
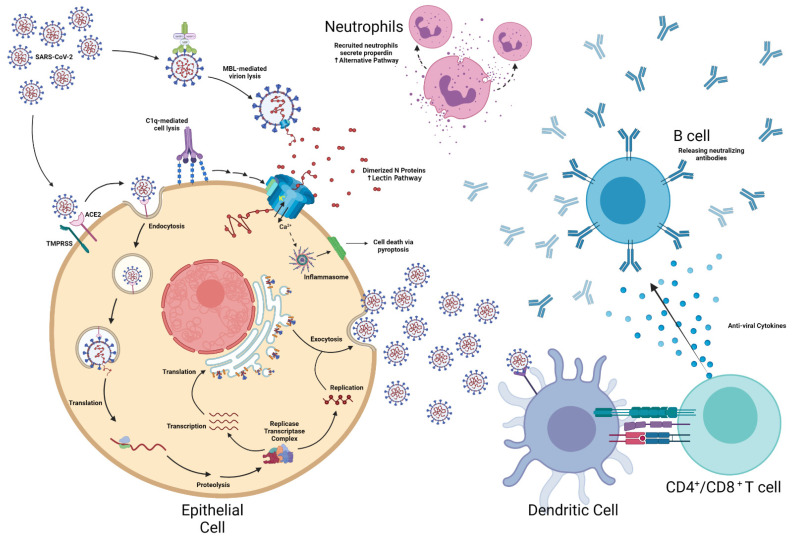
The immunological Impact of SARS-CoV-2 Infection [35,36,37,38,39,40,41]. Graphical contents were created with BioRender.com.

Concurrent to complement and inflammasome activation, antigen-presenting cells or virally infected cells stimulate both CD4^+^ and CD8^+^ T cells [30]. The activated T cells subsequently secrete anti-viral cytokines that prime B cells to release pathogen-neutralizing antibodies (Figure 1) [31]. The resulting immunoglobulins attempt to neutralize infection via phagocytosis; complement-mediated lysis; and/or antibody-dependent cellular toxicity [32]. Additional studies have also indicated the presence of cross-reactive antibodies in both healthy individuals and COVID-19 patients [16]. It is surmised that these antibodies were generated from previous coronavirus exposures, such as the common cold or the 2003 SARS-CoV-1 outbreak [16]. Correspondingly, researchers speculate that this may confer advantage against acute illness, but this has yet to be seen [16]. A possible mechanism for SARS-CoV-2 immune evasion is antibody-dependent enhancement (ADE) [16]. According to researchers, defective anti-spike immunoglobulins can facilitate Fc-receptor-mediated endocytosis of the coronavirus [16]. This would compel viral replication and systemic inflammation, resulting in a bleaker disease prognosis [16]. Fortunately, postliminary studies have indicated that ADE is unlikely the cause for severe reinfections [16].

Another humoral threat precipitated by COVID-19 infection is the advent of IFN autoantibodies [18]. Healthy individuals typically have a modest number of autoantibodies within their sera [18]. However, forgoing laboratory analyses have indicated the elevation of autoantibodies within infected individuals [18]. Anti-IFN immunoglobulin are more deleterious than cytokine storms because their damage is targeted, protracted, and pernicious [18]. Typically, these autoantibodies target proteins within the heart, brain, and blood vessels [18]. However, the most alarming targets are annexin A2 and phospholipids [18]. Annexin A2 is a pleotropic protein that notably maintains the integrity of the cellular membrane and blood vessels [18]. While phospholipids predominately modulate blood coagulation [18]. The functionality of these molecules is compromised in the interim of infection, which substantiates the notion of COVID-induced autoantibodies [18]. Furthermore, the development of autoantibodies is dilatory, which may account for the latency of severe symptoms [18]. Interestingly, males generally have more IFN autoantibodies when compared to females and, as a result, experience more severe infections [18].

Despite these irregularities, the humoral immune response does supply lasting immunity. In a cohort study examining the plasma of 200 recovering COVID-19 patients, 98% of them displayed lasting immunity with steady levels of anti-spike immunoglobulins, CD4^+^ T cells, and (to a smaller extent) CD8^+^ T cells [33,34]. The composition of immune cells varied among study participants [33,34]. However, the presence of these cells remained steady 6–8 months post infection, with only a modest decrease [33,34].

### 2.4. The Role of SARS-CoV-2 in Heterologous Immunity

Heterologous immunity occurs when infectious agents engender cross-reactive effector or memory T cells [42]. According to proteomic analyses, SARS-CoV-2 contains an assortment of MHC class I epitope pairs that are extremely cross-reactive and, thus, able to manipulate T cell-mediated immunity [43]. This heterologous immune response can either alleviate COVID-induced hypercytokinema or hinder viral clearance [43]. Auspiciously, researchers presume that SARS-CoV-2 cross-reactive epitopes mitigate the robust type I immune response and, thus, confer a significant advantage [43]. Interestingly, several cohort studies have also indicated that COVID-19 patients with atopic asthma tend to have milder disease courses compared to patients without asthma or with asthma not related to allergies [43]. In fact, on average, pediatric patients with atopic asthma had significantly less *ACE2* expression within their nasal epithelium compared to patients without asthma or non-allergy related asthma [43]. This insinuates that the former group developed memory T regulatory cells that can interact with the heterologous SARS-CoV-2 epitopes and quickly resolve COVID-induced hypercytokinemia [43]. Moreover, this also implies that there is an inverse relationship between T cell activation and COVID-19 severity [43].

### 2.5. SARS-CoV-2 Variants with an Increased Immune Evasion Capacity

All of the SARS-CoV-2 variants engender COVID-19 infection, however, virulence, diagnosability, and treatability may vary among viral strains [44]. At the time of this publication, the WHO was monitoring the variants listed in Table 1. Variants of concern (VOC) significantly changed the course of COVID-19 epidemiology; possessed increased virulence or transmissibility; and remained elusive to public health measures [44]. Variants of interest (VOI) are strains that recently experienced advantageous mutations that may increase virulence, transmissibility, immune evasion, and treatability [44]. Many countries have experienced the impact of VOI strains [44]. Additionally, these variants may endow VOCs [44]. The final designation, alert, signifies strains that have the potential to present a global risk [44]. However, phenotyping or epidemiological impact has yet to be seen [44]. The latter category requires close monitoring and at any time can escalate to a status of VOI or VOC [44]. There are currently two variants classified as a VOC by the United States: Omicron and Delta. As of 8 December 2021, a few confirmed cases linked to the Omicron variant have been detected in the United States. Meanwhile, Delta continues to be the predominant circulating variant [44].

### 2.6. Boosting Humoral Immunity with Currently Available COVID-19 Vaccines

SARS-CoV-2 N proteins are highly immunogenic and ubiquitously expressed during infection [45]. In consequence, scientist tried to exploit N protein biochemistry for vaccines and serological assay development [45]. Unfortunately, preliminary testing indicated that vaccines expressing N proteins exacerbated viral-induced pneumonia and enhanced associated respiratory diseases [46]. Interestingly, serological diagnosis using N protein antibody detection seemed to be very accurate in detecting early infections [45]. Nevertheless, the majority of vaccines approved by the United States Food and Drug Administration (FDA) are mRNA-based vaccines [47,48].

When writing this manuscript, two mRNA vaccines and one vector vaccine were commercially available in the U.S [47,48]. The Pfizer-BioNTech and Moderna vaccines are mRNA based, while the Janssen/Johnson & Johnson vaccine is vector based (Table 2) [49]. The mRNA vaccines typically elicited a higher neutralizing antibody titer when compared to patients that received convalescent plasma [46]. Moreover, the mRNA vaccines elicited a greater T cell response, which corresponded to a 90% efficacy rate in preventing COVID-19 during stage III clinical trials [46,47,48]. Likewise, the vector-based vaccine also elicited protective immunity by increasing the quantity of neutralizing antibodies [46]. However, on 12 April 2021, the United States Center for Disease Control (CDC) paused administration of the Johnson & Johns (Janssen) vaccine out of an abundance of caution [50].

According to a joint statement released by the CDC and FDA, six out of 6.8 million people who received the Johnson & Johnson vaccine developed a rare but serious condition known as vaccine-induced immune thrombocytopenia and thrombosis (VITT) [50,51,52,53]. VITT is a clotting condition in which patients develop autoantibodies to platelet factor 4 (PF4), resulting in aberrant platelet activation [51,52,53]. VITT typically occurs in the absence of heparin [51]. The diagnostic criteria for VITT include the presence of thrombosis; thrombocytopenia; D-dimer levels greater than 4000 FEU; and a high titer of platelet-activating anti-platelet factor 4 antibodies within the patients’ serum [51]. An individual must meet all five criteria to have a definite diagnosis of VITT, otherwise, additional evaluation by an expert hematology panel may be required [51]. Furthermore, no sex preponderance for the development of VITT has been reported [51]. Nevertheless, the individuals identified as having definite VITT by the CDC and FDA in there 2021 evaluation were all females between the ages of 18 and 48 that experienced disease manifestation 6–13 days following vaccination [50].

Interestingly, clinicians were not able to give these patients heparin to treat their blood clots, so alternative treatments, such as direct and indirect oral Xa inhibitors and direct thrombin inhibitors, had to be given [50,51]. The CDC and FDA advised individuals experiencing severe headaches, leg pain, abdominal pain, and shortness of breath 3 weeks postliminary of receiving the Johnson & Johnson vaccination should contact their health provider [50]. As of 23 April 2021, the CDC and FDA authorized the resumption of the Johnson & Johnson COVID-19 vaccination with a special indication that women younger than 50 years of age should consult their healthcare provider before taking this vaccine [51].

It is important to note that all of the listed vaccines elicit side effects including fever, chills, myalgia, headache, nausea, general unwellness, swollen lymph nodes, joint pain, and injection site pain [49]. Individuals who have experienced prior allergic reactions to vaccinations or injectable medications should consult their healthcare provider before receiving any of the COVID-19 vaccinations [49].

## 3. Renal Complications

During an active infection, the kidneys filter cytokines and virulence factors out of the blood, while resident dendritic cells concurrently present SARS-CoV-2 antigens to T cells within the renal draining lymph nodes [24,54]. This functioning seems efficacious, but processing blood-borne pathogens exposes the kidneys to reactive oxygen species, SARS-CoV-2 virions, and itinerate properdin, which engenders bystander complement activation and direct renal cell invasion [24,54,55,56].

When contaminated blood moves from circulation into the proximal tubules, luminal epithelial cells ingest and, subsequently, degrade cellular debris and circulating cytokines [57]. Interestingly, properdin evade degradation by binding to heparan sulfate moieties on the tubular brush border, therein eliciting robust complement activation via the alternative pathway [56]. Various sections of the kidney contain endogenous complement regulator proteins that should theoretically block resulting MAC formation [24]. However, SAR-CoV-2 virions engenders excess complement activation, which overwhelms both membrane-bound and fluid-phase complement regulators [24,54].

Additionally, when SARS-CoV-2 infiltrates tubular renal cells, nephropathies such as anti-glomerular basement membrane (anti-GBM) disease and membranous glomerulonephritis may occur [24,54]. Both of these pathologies result in the defacement of foot processes, podocytes detachment from the basement membrane, and collapsing glomerulopathy [54,55,57]. Moreover, repeated injury to the epithelial and interstitial cells will incite fibrosis, which latterly leads to acute renal failure [24,54].

Other nephropathies associated with systemic SARS-CoV-2 infection include ischaemia-reperfusion injury; rhabdomyolysis-associated tubular toxicity; cardiorenal syndrome (such as renal hypoperfusion, hypotension, and venous congestion); and nephrotoxic drug interactions [24,58]. According to some reports, COVID-19 patients receiving antibiotic or anti-viral medications are also at an increased risk for experiencing acute kidney injury (AKI) [58]. To that end, COVID-associated AKIs or pre-existing renal disorders increase the likelihood of having poorer clinical outcomes [19].

In one study investigating the renal function of 5449 hospitalized patients, approximately 36.6% of monitored patients experienced AKIs, and of that group, roughly 35% of them had higher mortality rates [20]. In their concluding remarks, the investigators noted that AKIs were more common in emergent COVID-19 patients and that laboratory reports indicating elevated baseline creatinine and urea, proteinuria, and hematuria were negative prognostic factor for survival [20]. Subsequently, the long-term impact of COVID-19 and its associated treatment regimens on renal function is an important undertaking that needs to be investigated further [19].

A novel approach to mitigate COVID-induced AKIs is continuous renal replacement therapy (CRRT) [19]. Moreover, SARS-CoV-2 viremia blocks the glomerular filtration barrier, which induces focal complement activation, therein eliciting tubulointerstitial fibrosis and glomerulosclerosis [19]. Unfortunately, fibrotic kidneys cannot maintain arterial blood pressure [19]. Subsequently, COVID-patients with chronic renal disease are highly susceptible to hemodynamic instability and instances of hypercoagulation [19]. These sequalae ultimately engender disseminated intravascular coagulation (DIC) and end organ dysfunction, which can be life threatening [19].

Fortunately, CCRT was successfully used to treat emergent patients during the SARS-CoV-1 and MERS-CoV epidemics [19]. In addition, COVID-19 patients placed on dialysis had fewer circulating lymphocytes, leukocytes, and pro-inflammatory cytokines than non-hemodialysis COVID patients [19]. This intervention correlated to milder symptoms and a lowered risk for developing ARDS [19]. However, reducing the presence of circulating immune factors may increase the risk of contracting other types of infection and lengthen COVID recovery times [19].

### 3.1. Recommended Guidelines for Kidney Transplantation Amid the SARS-CoV-2 Pandemic

Kidney transplantation should be executed on a case-by-case basis [19]. However, the general rules of thumb are as follows. According to Benedetti et al., living donor transplants should be suspended in areas with a high incidence of COVID-19 infections [19]. Likewise, if either the donor or the recipient resided in COVID hot spot areas within 14 days of the operation, then surgery should also be postponed [19]. In areas with sporadic COVID-19 cases, deceased donor transplants are permissible so long as the donor kidney is confirmed to be SARS-CoV-2 negative [19]. However, in areas with widespread infection, deceased donor transplants should be suspended [19]. And finally, transplants requiring T or B cell depletion should be deferred indefinitely even in places where the incidence of COVID-19 infection is considerably low [19].

Concerning the concomitant use of anti-rejection medications, the European Renal Association—European Dialysis and Transplant Association (ERA-EDTA) advises COVID-19 patients without pneumonia to continue taking their prescribed anti-rejection medication but at a reduced dosage [19]. In contrast, emergent patients should wean off their immunosuppressant by taking hydrocortisone/solumedrol [19]. Unfortunately, discontinuing anti-rejection medications may cause hypercytokinemia and kidney rejection [19]. Therefore, the latter approach should be exercised with caution [19].

Other important things to consider are drug-drug interactions between the anti-virals, anti-inflammatories, and anti-rejection medications [19]. Drugs used to treat COVID-19 may decrease the half-life of the immunosuppressants; therefore, clinicians are advised to monitor any subtle changes in creatine levels or estimated glomerular filtration rates and adjust accordingly [19].

### 3.2. COVID-19 Critical Care and the Associated Strain on CRRT Resources

Unfortunately, the critical care demand for adults with COVID-19 has put a significant strain on CRRT resources [59]. Now more than ever, adults with no prior AKIs are being placed on dialysis due to insidious viral-induced nephropathies [57]. Even during post-recovery, COVID-19 patients are at an increased risk for developing progressive chronic kidney disease [19]. So, in order to allocate resources more appropriately, the American Society of Nephrology (ASN) and pCRRT foundation recommend early diagnosis and prevention of AKIs; allocating one CRRT machine for multiple patients for shorter run times; limiting CRRT consumables; considering the regular use of anticoagulants; limiting infusion pumps; and utilizing alternative CRRT methods (e.g., peritoneal dialysis, routine hemadsorption, and IHD in hemodynamically stable patients) [59]. The efficacy of these alternative strategies has yet to be seen, however, it is something worth investigating in the near future [59].

## 4. Cardiovascular Complications

SARS-CoV-2 virions may have an increased predilection for the heart, as opposed to the lungs, due to the increased expression of *ACE2* and cathepsins B/L within cardiomyocytes, fibroblast, and pericytes [60,61]. Synergism between ACE2 and cathepsins B/L facilitates viral dissemination; with the role of cathepsins B/L being to prime SARS-CoV-2 for host membrane fusion [60]. Interestingly, cardiac cells display a positive correlation between cathepsins B/L presence and disease progression [17,62]. However, the role of ACE2 in cardiac pathogenicity appears to be much more complicated.

It is incontrovertible that SARS-CoV-2 uses ACE2 as a way to establish and promote infection [63]. However, forgoing research also indicates that SARS-CoV-2 concurrently downregulates *ACE2* expression in cardiomyocytes [63]. These findings may seem counterintuitive; nevertheless, several mouse models have confirmed that ACE2 levels are significantly reduced in cardiomyocytes following SARS-CoV-2 infection [60]. Likewise, in a similar study, researchers have also observed an increase in atherosclerotic plaque buildup; expression of adhesive molecules; and pro-inflammatory cytokine release in ACE2^−/−^ApoE^−/−^ mice when compared to ApoE^−/−^ mice [60]. Collectively, these contrivances can affect the neurohumoral system resulting in defective contractility and other significant cardiac morbidities [60,63,64]. Additionally, COVID-19 patients with elevated troponin, hypertension, atherothrombotic events, myocardial injury, hypercytokinemia, or who have recently received a heart transplant are at an increased risk of contracting a more severe infection that may lead to death [60,63,64].

### 4.1. Carditis

Under normal physiological circumstances, ACE2 mitigates pernicious hypertension by inducing the conversion of angiotensin II to angiotensin I [64]. In interim of COVID-19 infection, this protective functioning is attenuated due to the decreased expression of *ACE2* within the heart [64]. As a result, angiotensin II accumulates and pro-inflammatory cytokines are released [63,64]. This peculiarity was evidenced in postmortem studies in which CD68^+^ macrophages and T cells were ubiquitous within the endocardium [61].

In like manner, clinical studies indicated that COVID-19 has the propensity to induce myocarditis in patients with no prior cardiac problems and exacerbate inflammation in patients previously diagnosed with cardiovascular disease (CVD) [64]. The latter point is evidenced by the increased levels of myocardial stress markers—including myoglobin, NT-proBNP, and creatine kinase—among COVID-19 patients [64]. Moreover, patients with CVD and a history of hypertension are at a higher risk for experiencing deleterious cytokine storms, which can devolve into inflammatory-induced heart failure [64]. One study indicated that COVID-induced carditis can also affect pediatric patients in the form of Kawasaki disease [60]. The participant pool was small, however, a majority of them experienced COVID-induced myocarditis, with one participant experiencing a giant coronary artery aneurysm [60].

### 4.2. Coagulopathy and Septic Shock

COVID-19 patients with comorbidities have a proclivity for developing coagulopathies that are precipitated by hypercytokinemia or prolonged immobilization [64]. The pericytes of these patients are extremely maladaptive in interim of disease [64]. As a result, they are at an increased risk for experiencing pro-thrombolytic events such as DIC and venous thrombosis; although a differential diagnosis of DIC is considered rare [60,63,64]. Biological indications for these coagulopathies include elevated factors I and VIII; elevated D-dimers; and a modest reduction in platelet counts [60]. These parameters can be measured by traditional laboratory testing or via the viscoelastic methods (VEMs; i.e thrombelastography and thromboelastometry) [65]. VEMs may be preferred over traditional laboratory testing due to its speed and measurement of whole blood coagulation in real-time, which is very decisive for critically ill patients [65,66]. Nevertheless, some parameters of in vivo clotting are immeasurable by VEMs, which may require secondary laboratory testing [66]. 

Not surprisingly, elevated D-dimers (i.e., >1 g/L) correlated with an increased risk of in-patient deaths due to aortic embolisms and DVT [64]. Subsequently, the suggested treatment regimen for these patients includes aggressive antivirals combined with oral anticoagulants; low molecular weight or unfractionated heparins; and mechanical prophylaxis [64]. The exact mechanism for COVID-induce coagulopathies has yet to be elucidated [61]. Nonetheless, researchers speculate that SARS-CoV-2 directly infects and kills the endothelial cells, resulting in increased basement membrane thrombogenicity [61].

The incidence of septic shock and organ dysfunction among COVID-19 patients seems to be higher than that of DIC [67]. However, a retrospective study of 21 SARS-CoV-2 related deaths showed that more than 70% of these cases were caused by DIC [68,69]. In a study of 138 COVID-19 patients, the risk of developing septic shock was 30.6% among ICU patients, and only 1% among non-ICU patients [70]. Another study of 99 COVID-19 patients reported that septic shock followed by multiple organ dysfunction syndrome was the cause of death among 17% of SARS-CoV-2 related death cases [67,71].

### 4.3. Myocardial Infarction

COVID-19 patients with severe illness are liable to develop micro embolisms that can dislodge existing coronary plaques [61]. As a result, arteries can become occluded and related tissues can become ischemic; thereby increasing the likelihood of incurring a type I myocardial infarction [61]. Alternatively, acute respiratory distress syndrome caused by SARS-CoV-2 can impede pulmonary gas exchange, resulting in hypoxemia and pernicious ischemia [64]. The aforesaid damage mimics a type I thrombolytic event, however, the absence of fatty streaks and calcified blockages suggests a differential diagnose of type II myocardial infarction [64]. These ischemic cardiomyocytes will, subsequently, lead to the accumulation of intracellular calcium, resulting in mitochondrial dysfunction and oxidative stress [64]. The totality of these events, in conjunction with viral-induced hypercytokinemia, will result in cell death [64]. The agglomerate of apoptosed cells will ultimately engender troponin effusion and BNP elevation, which will provoke an extempore heart attack [64].

### 4.4. Arrhythmias

Heart palpitations are an important manifestation of SARS-CoV-2 infection in patients lacking cough or fever [60]. Documented arrhythmias include tachycardia, presyncope, bradycardia, a third heart sound, and tachypnea [63]. The corresponding electrocardiograms may indicate diffuse ST segments with peculiar concave morphologies; inversion of the T wave; ST segment elevation; hypokinesia; or hyperkinesia with apical ballooning [63].

Other documented arrhythmias include Takotsubo cardiomyopathy and Fulminant myocarditis [63]. Takotsubo cardiomyopathy can be induced by the physical or emotional tolls associated with the pandemic [63]. This myopathy occurs extempore but is, nonetheless, considered temporary [63]. Fulminant myocarditis, notwithstanding, is caused by hypercytokinemia-induced cardiogenic shock [63]. At the onset of systemic septicemia, both the atria and ventricles become desynchronized, resulting in an extempore heart attack and death [63]. This myopathy typically occurs in emergent SARS-CoV-2 patients and requires immediate attention and treatment [63]. Unfortunately, cardiac recovery from the previously mentioned arrhythmias is highly ineffectual due to the decrease expression of *ACE2* [63]. This lack of mitigation measures can result in permanent cardiac injury, dysfunction, or even death [63].

### 4.5. Heart Failure and Sudden Cardiac Arrest

Inconsistent respiration, in conjunction with neoteric arrhythmias and hypercytokinemia, provokes cardiac stress and insidious fibrosis [61]. The amassed fibrotic tissue gradually impedes the capacity of the heart to contract until it engenders viral induced heart failure [61]. Alternatively, coagulopathies precipitated by COVID-19 can also induce multi-organ failure, which subsumes heart failure [61].

Sudden cardiac arrest has been reported in 11% of hospitalized COVID-19 patients in a study involving 99 subjects with or without history of ischemic heart disease at early stages of the pandemic [67,70]. Another study investigated the role of SARS-CoV-2 infection in causing cardiac arrest among 1080 hospitalized and 1946 non-hospitalized patients [72]. COVID-19 was involved in 10% of non-hospitalized and 16% of hospitalized cardiac arrest cases [72]. Additionally, 30-day mortality rate increased in COVID-19 non-hospitalized and hospitalized patients by 3.4-fold and 2.3-fold, respectively [72]. These results imply that the cause of death could be due to an imbalance of pulmonary ventilation-perfusion ratio and a reduced pulmonary vasculature capacity [67]. Additional pathophysiological factors may include decreased level of functional residual gas resulting from occlusion of microvasculature, which subsequently leads to the development of pulmonary hypertension and cor pulmonale [67].

The direct role of SARS-CoV-2 infection in causing heart failure and sudden cardiac arrest needs further investigation. However, compelling preliminary data suggests that viral systemic inflammation disrupts the coronary microcirculation, resulting in myocardial ischemia [67]. Therefore, appropriate measures must be performed to reduce risk of death among COVID-19 patients, especially those with underlying cardiovascular conditions.

### 4.6. The Interconnecting Effects of COVID-19 Experimental Treatments and Heart Medications

The use of angiotensin-converting enzyme (ACE) inhibitors and angiotensin receptor blockers (ARB) in COVID-19 patients was initially very controversial [73]. On the one hand, burgeoning animal studies contended that ACE inhibitors and ARBs contribute to SARS-CoV-2 pathogenicity, by upregulating ACE2 receptors, and therefore should be replaced [73]. On the other, affluent scientific societies argued that these studies were very limited and may jeopardize the wellbeing of high-risk cardiovascular patients [73]. A timely research article subsequently revealed that neither ACE inhibitors nor ARBs affect *ACE2* expression directly [60]. Interestingly, the concurrent use of ACE inhibitors and ARBs corresponded with a milder inflammatory profile when compared to patients not receiving ACE inhibitors or ARBs [63]. This has led some researchers to postulate if concurrent use of ACE inhibitors and ARBs may attenuate SARS-CoV-2-indued cytokine storms, resulting in better patient outcomes [63]. However, this connection has yet to be determined in clinical trials [63].

Experimental treatments of SARS-CoV-2 infection with known cardiac contraindications include azithromycin, hydroxychloroquine, macrolides, lopinavir/ritonavir, and ribavirin [63,67]. Azithromycin and hydroxychloroquine increase the risk of serious arrhythmias and prolonged QT intervals, whereas macrolides can induce reoccurring ventricular extrasystoles or torsades des pointes [63,74]. Ribavirin can cause adverse endocrine and CNS reactions, and lopinavir/ritonavir can induce hypercholesterolemia and endocrine adverse reactions [74]. In view of these cardiac complications, it is important for clinicians to remain vigilant in documenting drug adverse effects and for patients to report them.

## 5. Endocrinological Complications

Neurological studies have indicated that SARS-CoV-2 infiltrates the brain via ACE2 receptors situated in the olfactory bulb [67]. The interplay between the olfactory system and the hypothalamus is well established [72,73]. To that end, researchers speculate that COVID-induced anosmia and ageusia precipitate adenoma formation; hormonal imbalances; and post viral syndromes such as fatigue, dizziness, and dejected mood [72,73,74,75]. These symptoms are multifarious and, in most cases, correlate to a poorer disease prognosis [75].

Correlative studies have also indicated that COVID-19 may engender a rare, but serious, autoimmune disorder known as lymphocytic hypophysitis [75,76]. Comorbidities associated with lymphocytic hypophysitis include diabetes insipidus and anterior pituitary hormone deficiencies [76]. If these maladies are left untreated, patients may develop pan-hypopituitarism, which can be life-threatening [76].

Unfortunately, there are conflicting views regarding the use of hormonal therapies in interim of coronavirus infection [72]. Those in favor of suspending treatment argue that select antiviral medications may contraindicate concomitant use of hormonal drugs [72]. For instance, some studies report that dopamine receptor agonists (DRAs) inappropriately interact with lopinavir/ritonavir, resulting in CYP3A4 enzyme inhibition; higher levels of plasma bromocriptine; and a proclivity to develop hypotension [72,77]. Another study indicated that concomitant use of DRAs with vasopressors might provoke vasospasms and a rapid rise in blood pressure [72]. These adverse events were predictably common in emergent COVID-19 patients [72]. However, patients with mild to moderate coronavirus infections may continue most hormonal treatments with limited to no repercussions [72]. Additionally, individuals with growth hormone (GH) deficiencies were advised to suspend replacement therapies until after their infection has cleared due to their increased susceptibility to develop adverse reactions [72].

### 5.1. The Impact of COVID-19 on the Adrenal Glands

Sepsis, hypotension, and hypoxia provoke activation of the hypothalamus–pituitary–adrenal gland (HPA) axis [72]. This induces the release of corticosteroids, which modulate the inflammatory immune response [72,78]. Unfortunately, forgoing studies have indicated that cortisol secretion is impeded amid critical illness, with some cases culminating in cortisol resistance [72]. This phenomenon is known as critical illness-related corticosteroid insufficiency (CIRCI) [72]. The pathogenesis of CIRCI involves the select decommissioning of glucocorticoid receptors, resulting in the exorbitant release of coagulation factors and inflammatory cytokines [72]. Thus, COVID patients with pre-existing adrenal insufficiencies or CIRCI are at risk for developing more aggressive respiratory tract infections, which can engender a fatal adrenal crisis [72]. To avert crisis, researchers recommend augmenting the adrenal insufficiency sick day treatment regimen [72]. For instance, Sultanian et al. advised to double the morning dose of hydrocortisone then administer four intermittent doses (20 mg each) throughout the day [72]. Unfortunately, concomitant use of hydrocortisone with ritonavir can result in adverse drug reactions, such as extending the half-life of hydrocortisone [72]. Protracted exposure to hydrocortisone will attenuate cytokine release, resulting in pestilent infections [72,79]. Therefore, the decision to deliver hydrocortisone treatment, in interim of coronavirus infection, should be made with great caution [72].

Other potential consequences for COVID-induced CIRCI include attenuation of immune cell extravasation; transient hypocortisolism; and poorer prognosis among patients with Cushing’s syndrome [80]. During the stress-induced immune response, norepinephrine, epinephrine, and cortisol induce immune cell trafficking [80]. However, CIRCI abates secretion of these hormones, theoretically causing an impairment of immune cell extravasation [80]. There is no direct evidence confirming this hypothesis, but it is something worth investigating in future studies [80]. Similarly, the effects of COVID-19 on patients with Cushing’s syndrome has yet to be elucidated [72]. Although comorbidities associated with Cushing’s syndrome including hypertension and diabetes, have been correlated with poorer disease prognosis [72]. Likewise, transient hypocortisolism may result in a poorer disease prognosis [72]. However, this malady has only been observed in SARS patients and has yet to be reported in COVID-19 patients [72].

Another important manifestation associated with coronavirus infection is electrolyte imbalance [72]. According to clinical observations, adrenocorticotropic hormone (ACTH) secretion is impaired in interim of disease, as a result, mineralocorticoid levels are depleted [72,75]. This condition is further exacerbated by GI disturbances, such as vomiting and diarrhea, which engenders both hypokalemia and electrolyte loss [72]. Collectively, these maladies foment the upregulation of RAAS, resulting in pernicious hypertension [72]. To circumvent this problem, Sultanian et al. recommend corticosteroids or desmopressin titration via intranasal, intravenous, or intramuscular administration [72]. Interestingly, some reports speculate that SARS-CoV-2 participates in molecular mimicry in which the virus mimics ACTH in order to evade immunodetection [74]. These reports go on to say that mimicking ACTH not only helps SARS-CoV-2 attenuate stress-induced cortisol surges, but it also tricks the nascent antibodies into attacking circulating ACTH instead of the virus itself [74]. This argument is very enticing because it has been confirmed that SARS-CoV-1 participates in the molecular mimicry of ACTH and that SARS-CoV-2 shares 95–100% protein homology with SARS-CoV-1 [74]. Yet, the ability of SAR-CoV-2 to mimic ACTH remains to be seen [72].

### 5.2. The Impact of COVID-19 on Adipose Tissue

The synergism between obesity and its associated comorbidities makes it difficult to discern the maladies specifically attributed to corpulence [80]. Nevertheless, forgoing studies have indicated that expression of *ACE2* and *TMPRSS2* within adipocytes is exorbitant [75]. This expounds the susceptibility of individuals with a higher body fat index to incur more onerous COVID-19 infections [80]. Moreover, visceral fat deposits can also secrete pro-inflammatory adipokines, which increases the likelihood of obese patients to develop hypercytokinemia and ARDS in interim of COVID-19 infection [74]. Unfortunately, the accumulation of visceral fat is also a comorbidity associated with aging and thus explains the poorer disease prognosis among this patient population [75].

Another potential adipose-associated virulence factor is dipeptidyl peptidase 4 (DPP4) [80]. DPP4 is a transmembrane protein that is ubiquitously expressed on adipocytes [80]. In addition, much like *ACE2* expression, *DPP4* expression can directly correlates to disease prognosis [80]. This conjecture, however, may be farcical because DPP4 typically interacts with MERS-CoV spike proteins, which share minimal sequence homology with SARS-CoV-2 [80,81,82]. In spite of this, clinical studies have indicated the utility of inhibiting DPP4, in interim of COVID-19 infection, to reduce inflammation [81]. The latter point suggests that the role of DPP4 in COVID-19 should be further investigated.

### 5.3. The Impact of COVID-19 on the Thyroid Gland

The most common thyroid manifestation associated with COVID-19 infection is euthyroid sick syndrome (ESS) [72]. ESS is a transient alteration of thyroid hormone secretion due to anterior pituitary insufficiency or viral-induced thyroiditis [72,75,83,84,85]. The pathogenesis of COVID-induced ESS involves the downregulation of the hypothalamus–pituitary–thyroid (HPT) axis, which foments low TSH, T3, and T4 secretion [72]. Generally, COVID-induced ESS occurs in emergent patients with acute or chronic illness [72,85]. Moreover, because of its semblance to central hypothyroidism, it may be difficult to differentially diagnose ESS [72]. Therefore, clinicians should re-evaluate signs and symptoms later on and avoid treatments involving thyroxine or liothyronine due to their limited safety and efficacy [72].

ESS-induced thyroid transmogrification has been documented in SARS patients [75]. In a postmortem study examining the thyroids of five SARS-CoV-1 patients, researchers observed significant destruction of the follicular and parafollicular thyroid cells, resulting in low T3 and T4 levels [75]. Destruction of the thyroid cells may expound COVID-induced hypothyroidism; however, current postmortem studies have neither confirmed nor denied this [75].

Unlike other endocrine disorders, individuals with pre-existing hypothyroidism were not inclined to experience COVID-induced hospitalizations [75]. These patients were advised to continue concomitant use of thyroxine, adjusting the dosage as needed [72]. Conversely, patients with pre-existing hyperthyroidism seemed to be at risk for developing contraindicative neutropenia [72]. According to reports, antithyroid medications may engender neutropenia, which mimics the signs and symptoms associated with COVID-19 [72]. Therefore, in order to differentially diagnose neutropenia, clinicians are advised to run a full blood count [72].

Unfortunately, thyroid functional panels are skewed amid acute illness [72]. Therefore, clinicians should remain vigilant in monitoring COVID patients with pre-existing thyroid conditions and make dose adjustments accordingly [72].

### 5.4. The Role of the Parathyroid Glands in COVID-19 Infection

Parathyroid hormone modulates vitamin D synthesis, which is important for mineral metabolism, physical barrier function, and innate/adaptive immunity [72,86]. In interim of COVID-19 infection, vitamin D is helpful because it reinforces the anti-inflammatory properties of the T_H_2 response, thereby mitigating COVID-induced hypercytokinemia [72]. Moreover, vitamin D also attenuates pro-inflammatory cytokine release from T_H_1 cells and modulates SARS-CoV-2 virulence by reducing the expression of its alleged functional receptor DPP4/CD26 [72].

Clinical studies have indicated that vitamin D deficiency engenders excessive calcium release, which may enhance viral replication [72]. According to molecular analysis, SARS-CoV-1 binds to calcium in vitro, thereby provoking a protein conformational change [72]. The relationship between calcium and SARS-CoV-2 has yet to be seen [72]. However, vitamin D deficiencies have been linked to ARDS and markedly onerous COVID-19 infections [72]. Unfortunately, the evidence expounding the efficacy of vitamin D supplementation has been mixed, so longitudinal studies may be needed [72].

### 5.5. The Potential Impact of COVID-19 on the Pancreas

*ACE2* expression is more abundant in the pancreas than in the lungs [79]. However, reports delineating the impact of SARS-CoV-2 infection on the pancreas are considerably uncommon [72]. The localization of ACE2 within the endocrine or exocrine pancreas is very controversial [72]. Even so, the prevalence of hyperglycemia and neoteric diabetes within the patient population suggests that SARS-CoV-1 preferentially infects β and δ cells [72]. Interestingly, SARS-CoV-1 infection increases the glycosylation of ACE2 and viral spike proteins, thereby enhancing its own virulence [72]. It remains to be seen if SARS-CoV-2 also infects endocrine islet cells [72]. Nevertheless, mild pancreatitis is an important malady observed in severe COVID-19 cases [72].

Researchers speculate that this sequela may be precipitated by viral invasion or systemic inflammation [72]. Unfortunately, clinical observations have explicitly concatenated pancreatitis with ARDS exacerbation [72]. This may expound the poorer disease prognosis of patients with this comorbidity [72]. Similarly, COVID-19 patients with pancreatic neuroendocrine tumors were also susceptible to severe infections due to their proclivity to develop thrombophilia and hyperglycemia [72].

### 5.6. The Correlation between COVID-19 and Diabetes Mellitus

SARS-CoV-2 may be an environmental trigger for both type I and type II diabetes mellitus [74]. According to foregoing research, SARS-CoV-1 foments cross-reactive antibody and T cell generation, thereby inducing β-cell destruction and type I diabetes [72]. Interestingly, SARS-CoV-1 can also induce type II diabetes by encouraging fetuin-A accrual [74]. The proclivity to develop either sequelae in interim of SARS-CoV-2 infection has yet to be determined [74]. However, previous reports have indicated that COVID-19 patients with pre-existing diabetes may experience worsening insulin resistance while taking lopinavir/ritonavir [74].

Unfortunately, animal studies have affirmed that diabetes mellitus increases the expression of ACE2 receptors in a variety of tissues, including the pancreas and lungs [72]. This explains why people with diabetes typically incur more severe COVID-19 infections [75]. Moreover, molecular analysis of the pancreatic β cells revealed ubiquitous expression of *Neuropilin 1* (*NRP1*) [75]. NRP1 is a membrane bound receptor that also facilitates SARS-CoV-2 entry [87,88]. Therefore, the omnipresence of NRP1 within β cells increases SARS-CoV-2 virulence and impedes insulin secretion, resulting in arduous infections and type I diabetes [75]. If left unchecked, COVID-induced type I diabetes can engender diabetic ketoacidosis, which can be fatal [75].

Another way COVID-19 can induce insulin resistance is by enhancing sympathetic activity [72,74,75]. According to antecedent studies, SARS-CoV-2 downregulates pulmonary ACE2 receptors, resulting in reduced angiotensin II degradation and increased aldosterone secretion [74]. The aforementioned process culminates in hypokalemia, which leads to uncontrollable glucose levels [74]. Furthermore, hypokalemia, in conjunction with basal inflammation, predisposes COVID-19 patients with pre-existing diabetes to ARDS, and thus, a poorer prognosis [74].

## 6. Gastrointestinal Complications

*ACE2* expression is 100 times greater in enterocytes when compared to the lungs [89]. Consequently, SARS-CoV-2 infection engenders tryptophan malabsorption, which results in decreased *Bifidobacterium* spp. and *Lactobacillus* spp. within the gut [89]. Surprisingly, a comorbidity of this transient interaction is intestinal inflammation, which, strangely enough, has no adverse effect on COVID-19 patients with inflammatory bowel disease [89]. The incipient manifestations of COVID-induced gastrointestinal (GI) problems include vomiting, diarrhea, abdominal pain, bleeding, a diminished appetite, or combination of the former [89,90,91,92,93]. In a multi-center cohort study evaluating the prevalence of COVID-induced GI manifestations, approximately 61% of the 318 subjects experienced GI symptoms [94,95]. These manifestations were also typified in pediatric patients with milder COVID-19 symptoms [90]. The pervasiveness of SARS-CoV-2 RNA within the enterocytes makes it subsistent within patients’ feces [88]. As a result, fomite transmission via toilet plume is another avenue for the spread of COVID-19 even in patients that are asymptomatic or recently recovered [89].

### 6.1. Stool Manifestations

Several studies have indicated that SARS-CoV-2 can be detected within the stool of serologically negative COVID-19 patients [89]. One study, examining the stool of 73 patients, indicated that 53.4% of them had SARS-CoV-2 positive stool 1–12 days following their initial diagnosis [90,91]. Moreover, 23.3% of the aforementioned cohort continued to test positive for SARS-CoV-2 RNA within their stool despite repeated negative respiratory results [90,91]. These findings were mirrored in pediatric patients in which 8 out of 10 children consecutively tested positive during rectal swabs despite having nasopharyngeal clearance [90,92]. Other related studies indicated that viral shedding could be detected in the stool 30 days following symptom resolution [90,93]. Interestingly, some researchers speculate that the presence of SARS-CoV-2 within the feces may induce a cytopathic effect, resulting in portal vein viremia [94].

### 6.2. Hepatic Involvement

Hepatocytes and cholangiocytes also have a high level of ACE2 receptors [94]. As a result, SARS-CoV-2 infection also foments liver dysfunction, resulting in elevated alanine aminotransferase and aspartate aminotransferase, which positively correlates to disease progression [89,90]. Most of the liver injuries are transient and mild; however, more severe injuries have been documented in the emergent patients [90]. Typically, COVID-19 patients with pre-existing liver injury or disease have a grimmer prognosis [89]. The mechanism for COVID-induced hepatic injury has yet to be elucidated [90]. However, forgoing studies suggest that immune-related injury, in conjunction with drug hepatotoxicity, may be attributed to this pathology [90].

Subsequent postmortem studies have uncovered microvascular steatosis with non-specific portal and lobular inflammation within liver biopsies, suggesting that the liver sustains substantial damage secondary to COVID-induced hypercytokinemia and hypoxia [89,94]. These manifestations were mirrored in patients with hepatotoxicity induced by lopinavir/ritonavir, remdesivir, tocilizumab, and to a lesser extent, azithromycin and hydroxychloroquine [94]. Complementary studies have suggested that the previously mentioned conditions correlate to a protracted hospital stay [89]. However, a multi-center cohort study should be performed to confirm this theory.

### 6.3. Impact on Gut Microbiome

The production of pro-inflammatory cytokines following SARS-CoV-2 infection may cause microbial dysbiosis, which can lead to systemic inflammation by triggering the release of microbial products and intestinal cytokines [96]. In order to develop novel therapeutic approaches, it is essential to investigate the microbiota interactions with cytokine responses in SARS-CoV-2 infection. Higher proteomic risk score (PRS) was associated with an increased risk of becoming critically ill among COVID-19 patients, which could be used as an indicator to predict the development of severe symptoms at early stages of infection [97]. Moreover, gut microbiome profiles and 20 blood-related proteins including C-reactive protein (CRP) were used to establish correlations with COVID-19 severity [97,98].

Healthy gut microbiota is rich in *Faecalibacterium* spp., *Bifidobacterium* spp., and *Ruminococcus* spp. [99]. Shotgun metagenomics sequencing analyses have shown that the abundance of *Coprobacillus*, *Clostridium ramosum*, and *Clostridium hathewayi* was positively associated with COVID-19 severe symptoms [98]. In contrast, an inverse correlation was observed between the abundance of the commensal microbe *Faecalibacterium prausnitzii* and infection severity [98]. An inverse correlation was also detected between *Bacteroides thetaiotaomicron* and SARS-CoV-2 fecal load among hospitalized COVID-19 patients, suggesting that certain microbiota-specific interventions, such as using probiotics, may serve as an effective way to reduce the development of COVID-19 severe symptoms [98].

## 7. The Impact of COVID-19 on the Reproductive System

Numerous studies have indicated that males typically experience poorer clinical outcomes than females [100,101]. Some estimates predict that males are 2.4 times more likely to contract and succumb to illness [101]. Subsequently, the biological bases for COVID-associated sexual dimorphism includes differences in allosome expression profiles; heterogeneity of sex hormone ratios; variations in gonadal *ACE2* expression; and divergent humoral immune responses [14,16,100,101,102].

Recent analyses have indicated that the gene for *ACE2*, the primary receptor for SARS-CoV-2 host entry, is localized to the X-chromosome [100,102]. Likewise, the gene for androgen receptors, which instigates *TMPRSS2* transcription and subsequent SARS-CoV-2 priming, is also localized to the X-chromosome [100,102]. Androgen is an omnipresent hormone used to maintain the male reproductive system [103]. Collectively, hemizygosity in conjunction with androgen production synergistically foment acute COVID-19 infections in males [100,102,104]. It is important to note that androgen levels steadily decrease with age [105]. However, age within itself is also a significant COVID-19 risk factor [100].

Concurrent immunological studies have also indicated that males typically inaugurate a less robust humoral immune response than females [100,101,102]. For the most part, males have a lower CD4/CD8 T cell ratio and a reduced type II immune response [101]. In fact, males frequently engender a type I immune response, which predisposes them to egregious sequelae such as hypercytokinemia and ARDS, both of which are infamously associated with a poorer disease prognosis [101]. In contrast, females are inclined to have a type II immune response, which is characterized by hefty toll-like receptor activation and anti-inflammatory cytokine release [101]. This response may be advantageous in interim of infection; however, it is decidedly duplicitous during preventative inoculations [106].

According to a prospective cohort study investigating the immunogenicity and reactogenicity of 131 pregnant and non-pregnant women following SARS-CoV-2 mRNA vaccination, 32% of pregnant women and 50% of non-pregnant women experienced notable adverse reactions [106]. Side effects included fevers, chills, injection site reactions, and malaise [106]. These maladies generally subsided a few days following the second dose, with no discernable long-term complications [106].

The final sexual dimorphic trait associated with COVID-19 is the partiality of *ACE2* expression within gonadal tissues [102]. According to recent reports, *ACE2* expression is exorbitant within the testes and sparse within the ovaries [102]. This abundance of ACE2 engenders orchitis, which may disrupt spermatogenesis [102]. Similarly, other studies have indicated that both germ cells and oocytes experience oxidative stress and subsequent apoptosis as a sequela of COVID-induced hypercytokinemia [102,107]. These quandaries pose a real threat to global fertility, and thus warrant further studying [102].

### 7.1. The Impact of COVID-19 on the Female Reproductive System

The role of estrogen in viral infections is well established in the literature [101]. According to forgoing research, estradiol can instigate the humoral immune response; modulate cellular migration to inflamed tissues; and bias T cell differentiation towards the T regulatory phenotype [101]. These functions will temper the host immune response and mitigate life-threatening sequela [101].

On a molecular level, estradiol also prevents SARS-CoV-2 priming and induces ACE2 ectodomain shedding by inhibiting the actions of TMPRSS2 and stimulating the actions of ADAM-17, respectively [101]. Solubilized ACE2 subsequently neutralizes unfettered SARS-CoV-2 [101]. Additionally, estradiol also inhibits the transcription of *DPP4*, which is another receptor used for SARS-CoV-2 host entry [101]. En masse, these actions decrease the rate of infectivity, which corresponds to shorter hospital stays and better clinical outcomes [101].

Unfortunately, estradiol is typically secreted by non-pregnant females of childbearing age [108]. Nevertheless, hormone replacement therapies, selective estrogen receptor modulators (e.g., tamoxifen and toremifene), and certain combinations of oral contraceptives (i.e., progestin-only pills) can also confer protection against viral-induced lung injury or ARDS [101]. In fact, recent reports have demonstrated that estrogen therapies can trigger the protein unfolding response and inhibit the activation of NLRP3 inflammasomes in interim of COVID-19 infection [101]. These actions protect host structures from sustaining significant inflammatory tissue damage, which also expedites recovery [101].

The distribution of *ACE2* expression within the female reproductive tract is generally low [109]. According to gross analyses, the vagina, endometrium, cervix, ovaries, and fallopian tubes all have low *ACE2* expression [109]. In spite of this, the trophectoderm and placenta appear to express higher *ACE2* and *TMPRSS2* levels during the course of pregnancy [109]. This insinuates that SARS-CoV-2 may be vertically transmitted from mother to fetus [109]. Ostensible subsidiary studies have also proposed that fetal receptors and key proteases, such as CD147, NRP1, and CTLS, are potential SARS-CoV-2 agonists, which help facilitate vertical transmission [100]. The veracity of these claims has yet to be elucidated, nevertheless, perspective cohort studies have auspiciously determined that vertical immunity, via placental transfer and breastfeeding, may occur following maternal vaccination [106,109].

For the most part, pregnancy outcomes are positive within COVID-19 patients [110]. Previously healthy women tend to experience milder symptoms with average preterm delivery rates and decidedly low neonatal and maternal mortality rates [109,110]. Women with pre-existing conditions, iatrogenic complications, and sudden decompensation, however, tend to have grimmer prognoses [109,110]. According to SARS-CoV-1 and MERS-CoV studies, ailing women were more likely to experience miscarriages, perinatal death, preeclampsia, and premature births [110]. This predisposition has not been affirmed in COVID-19 patients, but there are more pregnant women being admitted to the intensive care unit for COVID-related issues than non-pregnant women [110]. Moreover, of those women, a small fraction of them delivered babies that were also positive for COVID-19 [109,110]. Unfortunately, these studies had major limitations, which included a small study population, lack of standardized prenatal surveillance, and publication bias [110]. To that end, there is a great need for larger multi-center cohort studies that investigate the teratogenic effects of COVID-19 in women experiencing all stages of pregnancy [107,110].

### 7.2. The Impact of COVID-19 on the Male Reproductive System

The incipient role of testosterone in viral infections appears to be extremely deleterious [101]. According to hepatitis B and C studies, testosterone suppresses T cell activity and IFN-γ production, which laterally suppresses the innate and cellular immune responses [109]. Yet, testosterone levels negatively correlate to disease progression [109]. Specifically, hospitalized males with moderate to severe COVID-19 infections tend to have considerably low testosterone levels and remarkably high luteinizing hormone levels [109]. This inversion can be attributed to hypogonadism, which is a common sequela associated with systemic illnesses [109].

Another male-specific sexually dimorphic trait is high *ACE2* expression within the seminiferous ducts, Sertoli cells, Leydig cells, and spermatogonia [102]. According to recent reports, testicular expression of *ACE2* is contingent upon age: males aged 30 have the highest expression of *ACE2* while males aged 60 and older have the lowest expression of *ACE2* [102]. In like manner, *TMPRSS2* was also expressed in spermatids and spermatogonia [107]. Curiously, ACE2-positive spermatogonia were found to be enriched with genes related to viral transmission and reproduction but depleted with genes associated with male gamete generation [107]. Likewise, ACE2-positive Leydig and Sertoli cells displayed increased expression of genes related to intracellular junctions and the immune response but concurrently showed decreased expression of genes related to reproduction and mitochondria [107]. Viremia seems to precipitate these epigenetics, which engenders orchitis and defective spermatogenesis [102,109].

According to postliminary SARS-CoV-1 research, orchitis disrupts spermatogenesis by inducing lipid peroxidation of the sperm membrane and DNA fragmentation, which ultimately engenders germ cell apoptosis [102]. These studies also posited that emotional distress exacerbates orchitis, thereby reducing the quality and motility of remaining sperm [102,109]. Other factors that may impede fertility include some antivirals used in the course of treating COVID-19 infection and disinfectants commonly used for cleaning.

According to pharmacological studies, ribavirin, a broad-spectrum antiviral drug, decreases sperm count and causes DNA fragmentation for up to 8 months following the cessation of treatment [102,107]. Ribavirin is also known to induce oxidative stress, which impairs spermatogenesis and reduces testosterone levels [102]. Similarly, glucocorticoids also adversely affect the blood–testis barrier [107]. According to those reports, glucocorticoids foment germ cell apoptosis and, as a result, clinicians recommend short-term use for patients with progressive deterioration [107]. Disinfectants associated with sperm abnormalities include chlorine, which is commonly found in bleach, and iodine [107]. Excessive exposure to chlorine may lead to sperm head abnormalities while excessive exposure to iodine can lead to decreased sperm density [107]. The teratogenic effects of COVID-19 are still being investigated, so it is important that the public stay vigilant and informed. Fortunately, developing reports are actively dispelling common COVID-19 misconceptions such as this viral infection being a sexually-transmitted disease [111].

### 7.3. The Impact of COVID-19 on Gender-Affirming Care

Navigating gender-affirming care has been especially challenging during this pandemic [112]. Gender affirming surgeries (GAS) have been postponed until further notice, causing severe gender dysphoria [112]. Stigmatization of transgender and non-binary conforming (TGNC) individuals foments social, emotional, and occupational impairment, which could lead to a decline in physical and mental health [112]. Logistically speaking, postponing GAS can also jeopardize insurance approvals, which may set their transition back even further [112]. In some cases, TGNCs may be trepidatious that insurance coverage for these procedures will be revoked [113]. However, in the meantime, clinicians suggest that TGNC individuals planning to receive GASs should stay in touch with their healthcare team via telehealth, stop smoking immediately, and lose weight if possible, to make recovery easier and continue to cultivate self-awareness so that when the healthcare restrictions have been lifted, they will be ready to undergo the procedure [113].

## 8. The Effects of COVID-19 on the Integumentary System

ACE2 receptors are also located in the cutaneous and subcutaneous membranes, including the sebaceous glands, eccrine glands, arrector pili muscles, and associated vessels [114,115]. As a result, SARS-CoV-2 viremia facilitates integument ACE2 binding, which may elicit the development of viral exanthems, vasculitis, and micro-thrombotic skin lesions [115].

COVID-induced exanthems include morbilliform or maculopapular rashes, erythema multiforme, angioedema or urticaria, and vesicular eruptions [115]. Morbilliform exanthems are the most common skin manifestation associated with COVID-19, which are self-limiting rose-colored papules and macules that tend to coalesce [115,116,117]. Erythema multiforme exanthems are also characterized by macules and papules [113]. However, they tend to have classic target lesions, which predominantly manifest on the distal extremities [115]. Urticarial rashes generally manifest on the trunk and limbs and are rarely associated with angioedema [117]. Finally, vesicular cutaneous eruptions are similar to chickenpox and are characterized by monomorphic vesicles [116]. Collectively, these dermatological disturbances may be attributed to mast cell degranulation or drug-induced hypersensitivity [115]. Certain medication, including hydroxychloroquine/chloroquine, azithromycin, remdesivir, and tocilizumab, have been known to cause cutaneous adverse reactions [118]. Moreover, COVID-induced exanthems are typically associated with a milder course of disease and generally require liberal moisturization, topical corticosteroids, or nonsedating antihistamine as mainstay treatment regimens [115,116].

COVID-induced vasculitis/micro-thrombotic lesions include sacral ulcerations, acral pseudo-chilblain lesions, livedo reticularis, and purpuric lesions [114,115]. For the most part, COVID-induced vasculitis is precipitated by complement deposition, resulting in angiitis and tissue ischemia [115]. Acral pseudo-chilblain lesions are usually seen in asymptomatic COVID-19 patients [117]. These lesions are characterized by violet discoloration, itching, burning, and blistering [116,117]. These lesion are self-limiting and generally resolve on their own [117]. Conversely, livedo racemose-like lesions and purpuric lesions may be associated with a more severe course of COVID-19 infection [117]. Livedo reticularis can be transient, mild, lace-like, symmetrical, or dusty patches with a pale center [117]. These lesions are the milder version of livedo racemose, which is characterized by large, irregular, asymmetrical annular lesions, which are associated with pernicious coagulopathies [117]. Similarly, purpuric lesions are also associated with a more severe clinical course of infection and are characterized by intertriginous and acral blisters that may devolve into necrotic ulcerative lesions [117]. In general, vasculitis lesions are painful and typically appear during the latter half of active disease [115]. Other COVID-related skin maladies include acanthosis, dyskeratotic and necrotic keratinocytes, parakeratosis, and dermatitis [114,116]. Additionally, it has been proposed that SARS-CoV-2 may reactivate the human herpes virus, thereby eliciting pityriasis rosea [115].

En masse, these manifestations may appear during the prodromal, active, or convalescent phases of infection, and will generally persist for 6 days [114,115,116]. A majority of lesions will heal on their own completely, however, sequela such as erythema, petechiae, pernio, urticaria, and retiform purpura have been known to cause residual effects [114,116]. Remarkably, skin manifestations may be the only sign of COVID-19 infection in otherwise asymptomatic people [114,116]. Therefore, clinicians are advised to also monitor nascent skin manifestations during diagnostic triaging [114,116].

Unfortunately, personal protective equipment (PPE) and excessive handwashing can also foment the development of nascent skin conditions [115,119]. In general, contact and atopic dermatitis, dyshidrotic eczema, pressure injuries or urticaria, pruritus, folliculitis, seborrheic dermatitis, and acne are associated with personal hygiene and PPE adornment [115,119]. The most commonly effected areas are the nose, cheeks, hands, and forehead [119]. Prolonged use of goggles and facemask are typically associated with pressure injuries, while excessive handwashing and glove wearing are generally associated with epidermal maceration and erosions [115,119]. Unfortunately, goggle and facemask-associated injuries seem unavoidable [119]. However, reapplying hydrating creams or lotions can easily mitigate dermatitis associated with frequent handwashing [115,119].

## 9. The Musculoskeletal Consequences of COVID-19

A substantial musculoskeletal burden has been reported by SARS-CoV-1 patients, especially among those who experienced moderate or severe infection [120,121,122]. Symptoms including bone, skeletal muscle, soft tissue, and joint disorders were compelling signs of musculoskeletal involvement [120,121,122]. In vitro experiments and computational pathology have shown that SARS-CoV-1 and SARS-CoV-2 infections share almost identical pathophysiological responses [123]. Therefore, musculoskeletal dysfunction has been a common sign among patients recovering from COVID-19 [123]. Additionally, prolonged ventilator intubation may trigger pro-inflammatory conditions that can potentially have an impact on muscle and bone integrity in severe cases of infection [124,125].

Computed tomography (CT), ultrasonography, and magnetic resonance imaging (MRI) are used to evaluate musculoskeletal complications resulting from SARS-CoV-2 infection [123,126]. Although myalgia is a common clinical manifestation among COVID-19 patients, other musculoskeletal findings such as synovitis and osteonecrosis were less frequently reported at early stages of the current pandemic [126]. As the number of COVID-19 cases has started to rise, we have seen more neuromuscular and rheumatologic complications related to SARS-CoV-2 infection [127]. Implementing both biochemical signaling and computational modeling studies can be useful for identifying musculoskeletal molecular targets and long-term consequences of SARS-CoV-2 infection [123].

### 9.1. Myalgia

In large cohort studies, muscle aches have been frequently reported in 11–50% of COVID-19 patients [128]. Myositis and rhabdomyolysis have been described both as a late complication and as a presenting symptom SARS-CoV-2 infection [128,129]. Moreover, SARS-CoV-2 infection was found to trigger necrotizing autoimmune myositis in rare cases [130]. In comparison to similar age healthy control, moderate to severe SARS-CoV-1 patients had a 13% decline in walking distance measured over 6 min, and 32% decline in their grip strength [131,132]. These data suggest that SARS-CoV-1 infection can affect muscular strength and endurance [131]. Muscle biopsies collected from deceased SARS-CoV-1 patients have revealed muscle fiber necrosis and immune cell infiltration [131]. Similarly, SARS-CoV-2 infection leads to high production of pro-inflammatory cytokines and C-reactive protein (CRP), which may contribute to muscle weakness and fatigue [133].

About 25–50% of symptomatic COVID-19 patients reported generalized weakness or myalgia [134]. Study reports suggested that there is no correlation between infection severity and muscle pain contingency [135]. However, in patients who had an abnormal lung CT imaging results, myalgia was used as an indication of their overall disease progress [135]. Elevated creatine kinase (CK) levels were found in 19% of 214 COVID-19 patients with an upper range of 12,216 U/L, which is much higher than elevated CK levels reported among severe cases of SARS-CoV-1 patients (609 U/L) [136]. Multiple neurological symptoms affecting motor control and muscle function were reported in up to 36% of COVID-19 patients [136]. In addition to the potential direct viral effect, routine use of corticosteroids to suppress the inflammatory response in hospitalized COVID-19 patients may result in muscle atrophy, which could impair patients’ recovery process [123].

### 9.2. Arthralgia

Although arthralgias are commonly reported in patients with COVID-19, less is known about the osseous manifestations of SARS-CoV-2 [137]. Arthralgia is usually combined with myalgia, which makes it challenging to measure its prevalence alone [137]. Decreasing osteoblast proliferation and differentiation are induced by CXCL10, IL-17, and TNF-a, which may result in a net reduction in bone mass density (BMD) [138]. Additionally, severe inflammatory response can lead to chondrolysis and tendinopathy, which may impair the normal biological activity of chondrocytes and tenocytes, resulting in impaired matrix remodeling and potential exacerbation of degenerative tendon disorders [139,140]. Systemic inflammatory state during SARS-CoV-2 infection could explain the potential impact of viral infection on bone integrity.

Reduced BMD was observed in patients with SARS-CoV-1, which was initially thought to be due to the extent and duration of corticosteroids treatment [141]. Collectively, corticosteroid use and SARS-CoV-2-induced coagulopathy may contribute to the development of osteonecrosis and osteoporosis [142]. In patients with pre-existing inflammatory rheumatic and musculoskeletal disorders (RMD), advanced age was a major risk factor for hospitalization [143]. Moderate to severe RMD disease activity was also an independent risk factor for hospitalization, warranting the importance of continuing adequate RMD treatment during the COVID-19 pandemic [143].

Several studies have reported multiple chronic rheumatologic conditions triggered by SARS-CoV-2 infection, including systemic lupus erythematosus (SLE), rheumatoid arthritis (RA), and psoriatic spondyloarthritis [144,145,146]. Interestingly, these inflammatory arthropathies may be triggered by SARS-CoV-2 infection even in patients with mild or no respiratory symptoms, which underlines the necessity of COVID-19 testing in order to establish a definite correlation [147]. Non-specific imaging findings of SARS-CoV-2-related arthritis include synovial enhancement on MRI scan and power Doppler signal on ultrasound [148].

## 10. The Neurological Complications of COVID-19

There is an increasing body of evidence linking SARS-CoV-2 (COVID-19) infection to a plethora of short-lived non-consequential symptoms and long-term nervous system manifestations with more detrimental prognosis [149]. In fact, more than 90% of COVID-19 sufferers reported neurological symptoms of various severity [149]. Headaches, gustatory and olfactory dysfunctions, dizziness, and confusion are some of the most reported mild symptoms. They tend to appear with the disease onset or even prior to any other symptoms and as of the latest reports show no lasting side effects [149,150]. More life-threatening manifestations, however, have also been reported, such like stroke, cerebral venous thrombosis, seizures, Guillain-Barre syndrome (GBS), Miller Fisher syndromes (MFS), meningitis, and acute myelitis to cite only a few [149,150].

Since SARS-CoV-2 utilizes ACE-2 as the main entry point to the cells, one can argue that all cells expressing the ACE-2 receptor are potential targets for the virus [12]. In the nervous system, smooth muscle brain vasculature and choroid plexus cells were shown to express this docking protein [150,151]. Other cells such as, pericytes, microglia, and neurons were negative for ACE-2, which would make them unlikely candidates for COVID-19 infection [150,152]. However, most recent reports indicate that SARS-CoV-2 can use non-canonical routes such as basigin (BSG; CD147) or neuropilin-1 (NRP1) as docking alternatives to invade cells that otherwise do not express *ACE-2* [153]. This finding, if any, drastically increases the infectivity candidates for this virus.

Although not fully understood yet, there are several hypotheses as to how this virus invades the nervous system. The first route is through systemic distribution, from the airways into the brain’s endothelial cells of the blood brain barrier or the cerebro-spinal fluid-blood barrier, thereof making its way into the CNS realm. The second route involves a retrograde discrimination through the olfactory cells/bulb, ultimately finding its way upward into the neurons [154]. A third potential gateway for COVID-19 into the brain is non-surprisingly through the gastro-intestinal tract due to the widespread expression of *ACE-2* in the intestinal lining or potentially using alternative docking mechanisms, as previously mentioned [155]. Another potential plausible way to affect the brain is through the dramatic cytokine storm, which reaches far beyond the point of infection, therefore, potentially infiltrating the brain and causing local encephalitis and other reported brain dysfunctions [156]. There are currently very limited clinical data associating neurological disturbances to direct COVID-19 virus invasion of the brain. Instead, most neurological manifestations associated with COVID-19 illness result from the host’s viral defense mechanisms, which seem to disrupt brain microcirculation, causing hypoxia and neuronal cell death [149,156].

Below, we provide a succinct summary of the nervous system manifestations associated with SARS-CoV-2 infection. It is important, however, to note that collected data remain incomplete and in certain instances conflicting, potentially due to the ever-evolving nature of virus and its widespread reach. In fact, multiple strains have emerged since COVID-19 was first declared a pandemic by the WHO back on 11 March 2020. From the Delta-B.1.617.2 variant, which was first identified in India, to the most recent Omicron-B.1.1.529, freshly out of South Africa [44,157]. These variants of concern are a mere reminder of the unstable and ever evolving nature of this disease. It is therefore primordial that this review be regarded in the time lapse in which it was written.

### 10.1. General Neurological Symptoms

These symptoms precede and most often are the first sign of COVID-19 diagnosis. They may occur several days before the onset of respiratory symptoms and can last beyond the clinical manifestation period of the disease [149].

#### 10.1.1. Anosmia/Ageusia

Gustatory and olfactory dysfunctions include a sudden loss of taste (Ageusia) and smell (Anosmia) [158,159,160]. They are reported as a frequent initial symptom in mild to severe COVID-19 and are increasingly used as a biomarker for the disease [158]. A recent study showed 59 of 60 patients hospitalized with COVID-19 were found to have olfactory dysfunction [159]. A larger study involving multiple European medical centers also showed more than 85% COVID-19 positive patients suffered gustatory and olfactory disturbances [160]. Preliminary results show olfactory epithelium but not olfactory neurons to express *ACE-2* [149,161]. Epithelial cells bind to the COVID-19 spike protein, causing epithelial cell death through apoptosis. These findings indicate that COVID-19 does not directly infect neurons but rather affects the function of supporting cells [161]. Since sensory neurons are not directly affected, one silver lining is that CoV-2 infection is most likely transient and will not permanently damage olfactory neural circuits.

#### 10.1.2. Headache

Headache is reported as the most common and primordial neurological manifestation associated with COVID-19 infection [160]. It can be subdivided into two distinct phases: an early moderate, more diffuse pain otherwise associated with any viral systemic infection in the initial 5 days, followed by a more robust headache characterized by neck stiffness, bilateral temporoparietal, periorbital and forehead regions pain [160,161]. Headache is a non-specific symptom associated with multiple other diseases, however, sudden onset of headaches with increasing intensity in patients that otherwise did not suffer recurring headaches is a common symptom associated with COVID-19 infection and should prompt isolation and testing [161].

#### 10.1.3. Loss of Balance (Dizziness), Delirium and Confusion

Affecting close to 10% of COVID-19 positive patients, these manifestations can be the only presenting symptoms of COVID-19 infection or be part of a more complex presentation of the disease [162]. Baig et al., for instance, postulated hypoxia, hypercoagulopathy, and inflammatory storm induced by the acute respiratory distress syndrome consecutive to COVID-19 invading the respiratory system, as possible causes for these otherwise unrelated manifestations [163].

### 10.2. Severe Neurological Complications

Although statistics vary between different reports, neurological complications account for more than 40% of the overall COVID-19 population and a striking 60% of hospitalized COVID-19 sufferers [164]. Furthermore, having a pre-existing neurological condition significantly increases mortality rate due to COVID-19 infection [165,166].

Neurological complications can be subdivided based on how COVID-19 infection induces them. Encephalitis, for example, results from a direct viral invasion to the CNS. Stoke and other encephalopathies, however, emerge as collateral damage to the pulmonary inflammatory response syndrome and its associated cytokine storm [149,164,165,166].

#### 10.2.1. Cerebrocardiovascular Complications

Stroke and less frequently cerebral venus (sinus) thrombosis (CVT) were reported as major complications of COVID-19 infection with a prevalence of 2.3% versus 0.3%, respectively [164]. Stroke results from venous and arterial wall endothelial cell dysfunction and death following COVID-19 invasion, therefore, leading to disturbed blood brain barrier [164]. Other reports show an interplay between increased inflammation, increased coagulopathy, and decreased anti-coagulant mechanisms as potential culprits for COVID-19-induced Stroke [149,164,165]. The overall incidence of CVT is estimated to be around 1.5/100,000 per year and the prothrombic cascade triggered by COVID-19 infection is strongly linked to CVT [166]. Symptoms associated with this disease include severe headaches, visual impairment, increased intracranial pressure, and seizures [166]. This cardiovascular manifestation is also linked to the most critically ill cohorts of COVID-19 [149,166].

#### 10.2.2. Guillain-Barré (GBS) and Miller Fisher Syndromes (MFS)

GBS is polyradiculoneuropathy associated with many infections including COVID-19. It presents with various neurological symptoms such as lower limb weakness and progressive tetraplegia [167,168]. MSF, on the other hand, is characterized by ophthalmoplegia and areflexia [169]. It is a variant of GBS and is also caused by a systemic infection. The pathophysiology of these diseases in relationship to COVID-19 infection is yet to be fully understood, however, both diseases were shown to positively respond to immunoglobulin therapy [170,171].

#### 10.2.3. Meningitis, Encephalitis and Acute Myelitis

These diseases result from inflammatory response to viral or bacterial infection and have recently been reported in association with COVID-19 diagnosis [149,172,173]. Reported inflammation can affect the protective membranes of the brain (meninges), causing meningitis; the brain itself (encephalitis); or the spinal cord (acute myelitis) [172,173,174,175]. These conditions initially present with more common symptoms such as fever, headache, new-onset seizure, or feet weakness, which eventually evolves into paraplegia [172,173,174,175]. Intriguingly, Some COVID-19 patients with meningoencephalitis showed positive SARS-CoV-2 in their CSF, while others were CSF-negative for the virus [173,175]. As we await further studies, the current reports may indicate a potential direct and undirect link between these neurological manifestations and COVID-19 diagnosis.

## 11. The Long-Term Effects of Post-Acute COVID-19 Syndrome (PACS)

Unfortunately, there are a subset of COVID-19 patients that experience persistent symptoms months after being infected with the SARS-CoV-2 virus [176]. These patients range from acute to asymptomatic and typically develop long hauler manifestations 4 weeks post infection [176]. By and large, women tend to experience PACS more than men [176]. And the most common PACS symptoms include malaise, fatigue, chronic rhinitis, insomnia, dysgeusia, palpitations, headaches, chills, and sore throat [176].

Another complication associated with PACS is myalgic encephalomyelitis/chronic fatigue syndrome (ME/CFS) [176]. ME/CFS is a neuroinflammatory condition that elicits severe fatigue, muscle pains, and post-exertional malaise [176]. Several researchers have indicated that the development of PACS and/or ME/CFS can be linked to host–pathogen interactions and other biological factors such as host microbiome and autoimmunity [176]. Consequently, a multidisciplinary team of virologist, immunologist, neurologists, cardiologist, pulmonologist, and practitioners of physical therapy and rehabilitation would have to collaborate on PACS patient care and an individualized treatment plan [176].

It is important to note that most viral and bacterial pathogens elicit the development of chronic symptoms within a subpopulation of the afflicted [176]. In a study conducted by the CDC in 1994 investigating the development of ME/CFS-like symptom in 233 SARS survivors, approximately 27.1% of the former patients developed chronic fatigue syndrome, which parallels the present patient population afflicted with PACS [176]. Due to the recent nature of this global outbreak, the long-term effects of PACS are still being monitored and investigated.

## 12. Conclusions

**Figure 2:**
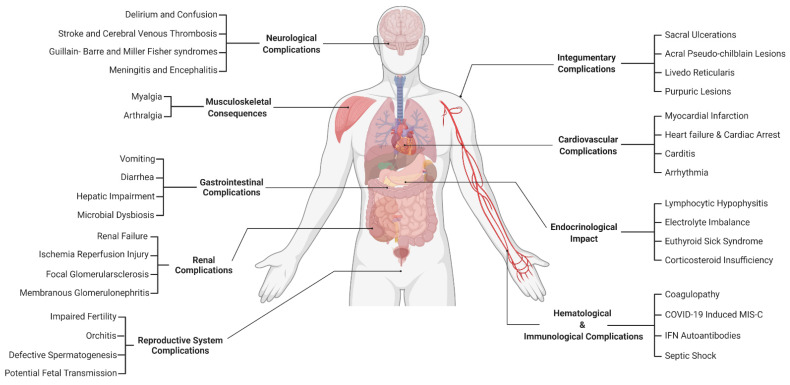
The potential extra-pulmonary complications of COVID-19 affecting multiple organ-systems [14,15,16,17,18,19,20,21,22,23,24,25,26,27,28,29,30,31,32,33,34,35,36,37,38,39,40,41,42,43,44,45,46,47,48,49,50,51,52,53,54,55,56,57,58,59,60,61,62,63,64,65,66,67,68,69,70,71,72,73,74,75,76,77,78,79,80,81,82,83,84,85,86,87,88,89,90,91,92,93,94,95,96,97,98,99,100,101,102,103,104,105,106,107,108,109,110,111,112,113,114,115,116,117,118,119,120,121,122,123,124,125,126,127,128,129,130,131,132,133,134,135,136,137,138,139,140,141,142,143,144,145,146,147,148,149,150,151,152,153,154,155,156,157,158,159,160,161,162,163,164,165,166,167,168,169,170,171,172,173,174,175,176]. Graphical contents were created with BioRender.com.

Over the last few decades, coronaviruses have been the origin of multiple highly infectious global outbreaks. The most recent coronavirus outbreak, SARS-CoV-2, developed in December 2019 and is characterized by systemic inflammation and acute hypoxic respiratory failure. Theoretically, any organ-system expressing the ACE2 receptor is susceptible to direct invasion by SARS-CoV-2, resulting in nonspecific or atypical symptoms. As the number of confirmed cases continues to rise, it is important to spread awareness of the extra-pulmonary manifestation of SARS-CoV-2 in order to expedite testing, diagnosis, and isolation (Figure 2).

SARS-CoV-2 enters the host via fomite exposure, respiratory droplets, and infective aerosols. The average incubation period of SARS-CoV-2 is approximately 2–14 days, with symptoms lasting for about 2 months. Once inside, SARS-CoV-2 colonization elicits aberrant complement activation, pro-inflammatory cytokine release, and the synthesis of other pyrogenic factors. Accordingly, inflammation is an essential prognosis factor for COVID-19 disease. Moreover, age, sex, and pre-existing co-morbidities can effect disease severity: males tend to develop more acute infections than females; the elderly are more susceptible to severe infections than younger populations; and pre-existing co-morbidities, such as diabetes mellitus or obesity, also increase the likelihood of contracting more onerous infections. Interestingly, SARS-CoV-2 contains an assortment of MHC class I epitope pairs, which elicits T-cell cross reactivity. In a host exposed to previous coronaviruses, this heterologous immune response may alleviate COVID-induced hypercytokinemia or hinder viral clearance. Nevertheless, foregoing research has indicated that there is an inverse relationship between T cell activation and COVID-19 severity.

During active infections, the kidneys filter cytokines and virulence factors out of the blood. Unfortunately, this exposes the kidneys to reactive oxygen species, SARS-CoV-2 virions, and itinerate properdin, which engenders bystander complement activation and direct renal cell invasion. Repeated injury to the epithelial and interstitial cells will incite ischemia-reperfusion injury and fibrosis, which latterly leads to acute renal failure. Fortunately, some researchers have discovered a novel approach to mitigate COVID-induced AKIs by treating severely ill patients with continuous renal replacement therapy. This therapy may reduce the amount of circulating cytokines but it also increases host susceptibility to opportunistic infections and lengthens COVID-19 recovery times.

In the cardiovascular system, COVID-induced hypercytokinemia engenders decreased *ACE2* expression within the heart, resulting in angiotensin II accumulation and the development of pernicious hypertension. The pro-inflammatory cytokine milieu also engenders defective contractility, development of nascent coagulopathies, and focal cardiac ischemia, which may devolve into either type I or type II myocardial infarctions. Unfortunately, many experimental treatments for SARS-CoV-2 infections have known cardiac contraindications. Therefore, it is important for clinicians to remain vigilant in documenting drug-adverse effects and for patients to report them.

Concerning the endocrine system, COVID-induced anosmia and ageusia may precipitate adenoma formation, resulting in multifarious hormonal imbalances. Cortisol secretion from the HPA axis can be impaired in emergent COVID-19 patient. This condition can elicit the exorbitant release of pro-inflammatory cytokines and coagulation factors, which contributes to the development of pernicious hypertension. Additionally, COVID-19 infection can be an environmental trigger for both type I and type II diabetes mellitus or exacerbate the condition of patients with pre-existing diabetes. In extremely rare cases, COVID-induced hypercytokinemia may engender an adrenal crisis or lymphocytic hypophysitis, both of which can be life threatening.

The impact of COVID-19 on the reproductive system is very polarized. Males typically experience poorer clinical outcomes due to a stronger type I immune response. Moreover, the increased *ACE2* expression within the testes and the deleterious role of testosterone in interim of infection impedes spermatogenesis and thus male infertility. Conversely, females tend to inaugurate a type II immune response due to the protective functioning of estrogen. Interestingly, both oocytes and germ cells may experience oxidative stress and subsequently apoptosis within the COVID-inflammatory milieu. However, several studies have indicated that pregnancy outcomes within previously healthy COVID-19 patients tends to be positive. Women with preexisting conditions, iatrogenic complications, and sudden decomposition, notwithstanding, tended to experience miscarriages, perinatal death, preeclampsia, and premature births. Unfortunately, the teratogenic effects of COVID-19 have yet to be elucidated and thus require further investigation.

In the integumentary system, SARS-CoV-2 viremia induces the development of viral exanthemas, vasculitis, and micro-thrombotic skin lesions. Some studies have indicated that SARS-CoV-2 can also reactivate the human herpes virus, thereby provoking the formation of pityriasis rosea. Integument manifestations may appear anytime during the infectious process and generally persist for about 6 days.

Other extra-pulmonary COVID-19 manifestations include transient intestinal inflammation, gut dysbiosis, mild hepatic injuries, and musculoskeletal dysfunctions. Prolonged illness may impact the structural integrity of the bones and muscles, resulting in decreased muscle tone and bone mass density. The potential for recovery from these maladies remains to be seen.

The emergence of the delta SARS-CoV-2 VoC has caused new waves of viral infection, due to its increased transmissibility and longer duration of infectiousness. At the time of this publication, the delta VoC remains the dominant VoC in many countries including the United States. On 25 November 2021, a new SARS-CoV-2 VoC, omicron, was detected. This VoC has emerged at a time when vaccine immunity is increasing worldwide, which makes it different from all previous VoCs emerging before vaccines were readily available. Preliminary epidemiological evidence suggests that omicron VoC seems to be highly transmissible, although it is not yet clear whether its transmissibility is higher than the delta variant. Early indications suggest that it is spreading quickly despite high levels of natural immunity to the delta variant. Therefore, omicron is anticipated to displace delta as the dominant VoC in several countries.

The majority of approved COVID-19 vaccines in the U.S are mRNA-based. These vaccines typically elicit higher neutralizing antibody titers when compared to patients that received convalescent plasma. Similarly, the FDA-approved vector-based virus also elicits protective immunity by increasing the quantity of neutralizing antibodies. The scientific community and governing bodies continue to optimize preventative care and treatments for COVID-19. Nevertheless, long-hauler symptoms may occur, resulting in chronic fatigue syndrome. We hope this paper will contribute to the larger body of knowledge by highlighting extra-pulmonary manifestations that have been documented to date.

## Figures and Tables

**Table 1:** SARS-CoV-2 variants being monitored by the WHO.

Variant Classification	WHO Label	Pango Lineages	GISAID Clade	Nextstrain Clade	Spike Protein Amino Acid Changes Being Monitored	Earliest Documented Samples	Date of Designation
**Variants of Concern**	Alpha	B.1.1.7	GRY	20I (V1)	+S:484K +S:452R	United Kingdom, September 2020	18 December 2020
	Beta	B.1.351 B.1.351.2 B.1.351.3	GH/501Y.V2	20H (V2)	+S:L18F	South Africa, May 2020	18 December 2020
	Gamma	P.1 P.1.1 P.1.2	GR/501Y.V3	20J (V3)	+S:681H	Brazil, November 2020	11 January 2021
	Delta	B.1.617.2	G/478K.V1	21A	+S:417N	India, October-2020	VOI: 4 April 2021 VOC: 11 May 2021
		AY.1					
		AY.2				Multiple countries,	VUM: 24 November 2021
	Omicron	B.1.1.529	GR/484A	21K	INS214EPE	November 2021	VOC: 26 November 2021
**Variants of Interest**	Eta	B.1.525	G/484K.V3	21D		Multiple countries, December 2020	17 March 2021
	Iota	B.1.526	GH/253G.V1	21F		United States of America, November 2020	24 March 2021
	Kappa	B.1.617.1	G/452R.V3	21B		India, October 2020	4 April 2021
	Lambda	C.37	GR/452Q.V1	21G		Peru, December 2020	14 June 2021
**Other Variants Currently Being Monitored**		B.1.427 B.1.429 *	GH/452R.V1	21C		United States of America, March 2020	VOI: 5 March 2021 Alert: 6 July 2021
		P.2 *	GR/484K.V2	20B/S.484K		Brazil, April 2020	VOI: 17 March 2021 Alert: 6 July 2021
		P.3 *	GR/1092K.V1	21E		Philippines, Jan-2021	VOI: 24 March 2021 Alert: 6 July 2021
		R.1 R.2	GR	-		Multiple countries, January 2021	7 April 2021
		B.1.466.2	GH	-		Indonesia, November 2020	28 April 2021
		B.1.621	GH	21H		Colombia, January 2021	26 May 2021
		AV.1	GR	-		United Kingdom, March 2021	26 May 2021
		B.1.1.318	GR	20B		Multiple countries, January 2021	2 June 2021
		B.1.1.519	GR	20B		Multiple countries, November 2021	2 June 2021
		AT.1	GR	-		Russian Federation, January 2021	9 June 2021
		C.36.3 C.36.3.1	GR	20D		Multiple countries, January 2021	16 June 2021
		B.1.214.2	G	-		Multiple countries, November 2020	30 June 2021

Data was obtained from the WHO Official Website [44].

**Table 2:** Current vaccines authorized in the U.S for the inoculation against COVID-19 infection.

Company	Vaccine Type	Efficacy against Preventing COVID-19 Symptoms	Number of Doses	Protection after First Dose?	Age
**Pfizer-BioNTech**	mRNA	95%	2 doses, 21 days apart	Limited	12 years and older
**Moderna**	mRNA	94%	2 doses, 28 days apart	Limited	18 years and older
**Janssen/Johnson & Johnson**	Vector	66%	1 dose	Some protection 2 weeks following vaccination	18 years or older

Data were obtained from the Mayo Foundation for Medical Education and Research [49].

## Data Availability

Not applicable.

## Abbreviations

ACE	Angiotensin Converting Enzyme
ACTH	Adrenocorticotropic Hormone
ADE	Antibody-dependent Enhancement
AKI	Acute Kidney Injury
Anti-GBM	Anti-glomerular Basement Membrane Disease
ARB	Angiotensin Receptor Blockers
ARDS	Acute Respiratory Distress Syndrome
ASN	American Society of Nephrology
BMD	Bone Mass Density
CDC	United States Center for Disease Control
CIRCI	Critical Illness-related Corticosteroid Insufficiency
CK	Creatine kinase
COVID-19	Coronavirus Disease 2019
CRRT	Continuous Renal Replacement Therapy
CSF	CerebroSpinal Fluid
CT	Computed Tomography
CVD	Cardiovascular Disease
CVT	Cerebral Venus (sinus) Thrombosis
DIC	Disseminated Intravascular Coagulation
DPP4	Dipeptidyl Peptidase 4
DRA	Dopamine Receptor Agonist
ERA-EDTA	European Renal Association- European Dialysis and Transplant Association
ESS	Euthyroid Sick Syndrome
FDA	United States Food and Drug Administration
GAS	Gender Affirming Surgeries
GBS	Guillain-Barré syndrome
GH	Growth Hormone
GI	Gastrointestinal
HPA	Hypothalamus-pituitary-adrenal gland axis
IFN/ IFN-γ	Interferon-gamma
IHD	Intermittent Hemodialysis
MAC	Membrane Attack Complex
MASP 1	MBP-associated serine protease 1
MASP 2	MBP-associated serine protease 2
MBP	Mannan-binding protein
ME/CFS	Myalgic Encephalomyelitis/Chronic Fatigue Syndrome
MERS-CoV	Middle East Respiratory Syndrome Coronavirus
MIS-C	Multi-system Inflammation Syndrome
MFS	Miller Fisher syndrome
MRI	Magnetic Resonance Imaging
NRP1	Neuropilin-1
N-protein	SARS-CoV-2 nucleocapsid protein
PACS	Post-acute COVID-19 Syndrome
PPE	Personal Protective Equipment
PRS	Proteomic Risk Score
RAAS	Renin Angiotensin Aldosterone System
RMD	Rheumatic and Musculoskeletal Disorders
SARS-CoV-1	Severe Acute Respiratory Syndrome Coronavirus 1
SARS-CoV-2	Severe Acute Respiratory Syndrome Coronavirus 2
S-protein	SARS-CoV-2 spike protein
TGNC	Transgender and non-conforming individuals
TLR	Toll-like Receptor
TMPRSS2	Transmembrane Serine Protease-2
VEM	Viscoelastic Methods
VITT	Vaccine-induced Immune Thrombocytopenia and Thrombosis
VOC	Variants of Concern
VOI	Variants of Interest
WHO	World Health Organization
